# Associations of life-course cardiovascular risk factors with late-life cerebral haemodynamics

**DOI:** 10.1177/0271678X241301261

**Published:** 2024-11-17

**Authors:** Mathijs BJ Dijsselhof, Jorina Holtrop, Sarah-Naomi James, Carole H Sudre, Kirsty Lu, Luigi Lorenzini, Lyduine E Collij, Catherine J Scott, Emily N Manning, David L Thomas, Marcus Richards, Alun D Hughes, David M Cash, Frederik Barkhof, Jonathan M Schott, Jan Petr, Henk JMM Mutsaerts

**Affiliations:** 1 Dept. of Radiology and Nuclear Medicine, Amsterdam University Medical Centers, Vrije Universiteit, NL; 2 Amsterdam Neuroscience, Brain Imaging 601873 , NL; 3 MRC Unit for Lifelong Health and Ageing at UCL, University College London, UK; 4 Centre for Medical Image Computing, Department of Computer Science, 4919 University College London, UK; 5 Department of Biomedical Computing, School of Biomedical Engineering & Imaging Sciences, King’s College London, UK; 6 Dementia Research Centre, Department of Neurodegenerative Disease, UCL Queen Square Institute of Neurology, University College London, London, UK; 7 Clinical Memory Research Unit (R.O.), Lund University, Sweden; 8 Institute of Nuclear Medicine, University College London Hospital NHS Foundation Trust, London, UK; 9 Neuroradiological Academic Unit, Department of Brain Repair and Rehabilitation, UCL Queen Square Institute of Neurology, London, UK; 10 UK Dementia Research Institute at University College London; 11 Queen Square Institute of Neurology and Centre for Medical Image Computing, University College London, UK; 12 Helmholtz-Zentrum Dresden-Rossendorf, Institute of Radiopharmaceutical Cancer Research, Dresden, DE

**Keywords:** Ageing, arterial spin labelling, cardiovascular risk factors, cerebral blood flow, cerebrovascular health

## Abstract

While the associations of mid-life cardiovascular risk factors with late-life white matter lesions (WMH) and cognitive decline have been established, the role of cerebral haemodynamics is unclear. We investigated the relation of late-life (69–71 years) arterial spin labelling (ASL) MRI-derived cerebral blood flow (CBF) with life-course cardiovascular risk factors (36–71 years) and late-life white matter hyperintensity (WMH) load in 282 cognitively healthy participants (52.8% female). Late-life (69–71 years) high systolic (B = −0.15) and diastolic (B = −0.25) blood pressure, and mean arterial pressure (B = −0.25) were associated with low grey matter (GM) CBF (p < 0.03), and white matter CBF (B = −0.25; B = −0.15; B = −0.13, p < 0.03, respectively). The association between systolic blood pressure and GM CBF differed between sexes (male/female B = −0.15/0.02, p = 0.04). No associations were found with early- or mid-life cardiovascular risk factors. Furthermore, WMHs were associated with cerebral haemodynamics but not cardiovascular risk factors. These findings suggest that cerebral blood flow autoregulation is able to maintain stable global cerebral haemodynamics until later in life. Future studies are encouraged to investigate why cardiovascular risk factors have differential effects on haemodynamics and WMH, and their implications for cognitive decline.

## Introduction

Mid-life cardiovascular risk factors are related to cerebrovascular lesions observed on MRI and are known to increase, by up to 40%, the risk of late-life cognitive decline.^1–3^ More specifically, the Framingham Heart Study (FHS) has shown that the cardiovascular risk score (FHS-CVS) and hypertension are associated with white matter hyperintensities (WMHs).^4,5^ WMHs are considered a common radiological marker of cerebrovascular pathology and are also linked to cognitive decline.^6,7^ However, WMHs detail end-stage cerebrovascular pathology and, once detected, allow for fewer opportunities to intervene through lifestyle changes or medication which might take up to 10 years to take effect.^8^ An earlier biomarker of cerebrovascular dysfunction, one based on haemodynamics, is needed to study the complex interplay between cardiovascular risk factors, cerebrovascular health, and the path to cognitive decline.

Arterial Spin Labelling (ASL) perfusion MRI is able to assess cerebral haemodynamics by quantifying cerebral blood flow (CBF).^9^ Adequate CBF is required to ensure the delivery of oxygen- and nutrient-dense blood to the brain tissue and is affected by cardiovascular risk factors.^10^ For example, high blood pressure (BP) may trigger cerebrovascular remodelling, which can alter global and regional CBF and ultimately may lead to WMHs.^11^ Literature on the relationship between CBF and cognition is conflicting, as regional increases and decreases in CBF are found in individuals at different stages of cognitive impairment.^12–15^

Furthermore, the spatial variation of ASL label arrival, quantified as the spatial coefficient of variation (sCoV), can indicate the efficiency of blood delivery from the main feeding vessels to distal brain tissue^16^ and is shown to gradually increase in individuals with more significant cognitive decline.^17^ These findings suggest that changes in cerebral haemodynamics might act as an intermediate step between cardiovascular risk factors and WMHs. Furthermore, assessment of cerebral haemodynamics may aid in predicting those at high risk of future cognitive decline, therapy monitoring, and choice of treatment strategy.

While these results are promising for lifestyle and medical treatment interventions to protect cerebrovascular health, some results are conflicting, and came from studies with either a cross-sectional design^14,18^ or with short follow-up times.^2,5,19,20^ A longitudinal life-span population study could allow investigating the impact of early-, mid-, and late-life cardiovascular risk factors on late-life cerebrovascular health more robustly, and in more detail. The British 1946 Birth cohort (the Medical Research Council (MRC) National Survey of Health and Development (NSHD))^21,22^ is a longitudinal cohort study with participants born in the same week of March 1946. These participants have been followed throughout their lives, assessing demographic, genetic, (cardio)vascular, and cognitive health at several visits. A subset participated in a neuroimaging substudy at 69–71 years old, referred to as the Insight 46 cohort. Whereas associations of cardiovascular risk factors with WM microstructural integrity^22^ and WMHs^21^ have been demonstrated in this cohort, it is still unclear how this relates to late-life cerebral haemodynamics such as CBF and macrovascular efficiency (operationalised by sCoV). The Insight 46 ASL perfusion MRI measurements provide a unique opportunity to investigate the impact of life-course cardiovascular risk factors on late-life cerebral haemodynamics (CBF and sCoV).

Therefore, with similar analyses to previous Insight 46 studies, we investigate the associations between 1) life-course cardiovascular risk factors and late-life cerebral haemodynamics, and 2) life-course cardiovascular risk factor changes between visits and late-life cerebral haemodynamics, 3) between late-life cerebral haemodynamics and WMH. Additionally, we investigated to what extent these associations are modified by sex. We hypothesise that worse cardiovascular risk factors, across the life-course, will be linked with reduced cerebral perfusion and macrovascular efficiency. Finally, we hypothesise that worse cerebral haemodynamics are associated with a higher WMH burden.

## Methods

### Study design

**Figure 1.**
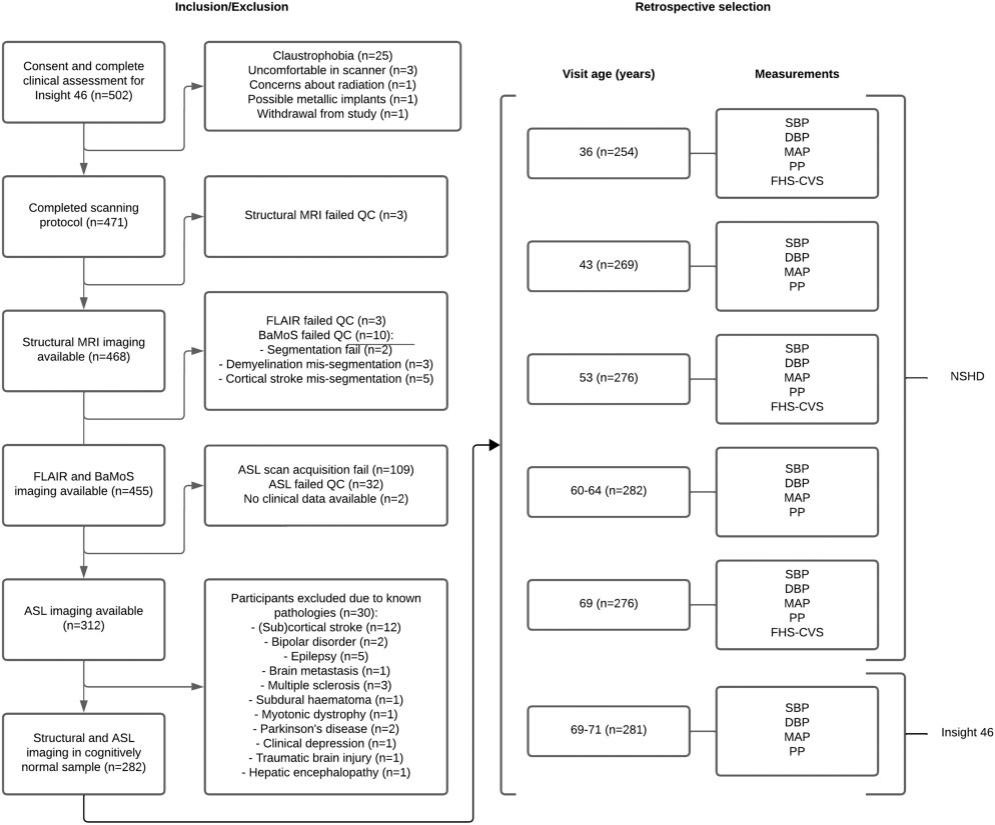
Flowchart of Insight 46 participant inclusion criteria and subsequent retrospective selection from the NSHD cohort. The numbers detail the number of participants included retrospectively at each visit. ASL: arterial spin labelling; BaMoS: Bayesian Model Selection; FLAIR: fluid-attenuated inversion recovery; QC: quality control.

Study participants were drawn from the Insight 46 cohort,^23^ a sub-study of the MRC NSHD; British 1946 birth cohort.^24^ At 36 and 43 years (early-life), 53 and 60–64 (mid-life), and 69 years of age (late-life), blood pressure measurement data were obtained from the NSHD parent cohort. At 36, 53, and 69 years, blood was drawn. At the additional Insight 46 neuroscience sub-study visit (69–71 years, late-life), neuroimaging was performed, and blood pressure measurement data was obtained between May 2015 and January 2018. Individuals with any contra-indications to MRI or Positron Emission Tomography (PET) imaging or missing relevant life course data were excluded, as described in the Insight 46 study protocol.^23^ Ethical approval for NSHD was obtained from the Greater Manchester Local Research Ethics Committee and the Scotland Research Ethics Committee. The National Research Ethics Service (NRES) Committee London granted ethical approval for Insight 46 (14/LO/1173). Research was conducted in accordance with the Helsinki declaration. All participants gave written informed consent. See Figure 1 for an overview of the data collection and participant inclusions.

### Life-course cardiovascular risk factors

The cardiovascular examinations included seated BP measurements taken at ages 36, 43, 53, 60-64, and 69 years, as well as standing BP measurements taken at 69–71 years (Insight 46 phase 1). Blood pressure measurements were previously described in detail.^21^ In short, two BP measurements were taken a minute apart after 5 minutes of rest while seated (NSHD visits, Figure 1) or lying (Insight 46 visit, Figure 1). The second BP measure was used for analyses unless missing. Conversion equations were applied to ensure compatibility between measuring devices were described previously.^21^ Systolic BP (SBP) exceeding 140 mmHg or diastolic BP (DBP) exceeding 90 mmHg were considered hypertension.^25^ Self-reported use of BP-lowering medication including diuretic medication, anti-hypertensive medication, nitrate vasodilators, or beta-blockers was registered, and SBP and DBP values used in this study were increased by 10 and 5 mmHg, respectively, if any BP-lowering medication were used.^26^ To calculate pulse pressure (PP), DBP was subtracted from SBP. Mean arterial pressure (MAP) was calculated by adding 1/3 PP to DBP.^27^

Because blood was drawn only at 36, 53, and 69 years of age, the required data to calculate FHS-CVS were only available for those visits. FHS-CVS was calculated for ages 36, 53, and 69 years by assigning risk points for age, sex, SBP, use of antihypertensive medication, diabetes mellitus type 2 history, current smoking, and body mass index (BMI; kg/m^2^).^28^ Smoking status was defined by postal questionnaires and diabetes mellitus status based on self-reported diagnosis or a haemoglobin A_1C_ level exceeding 6.5%.^29^

### Imaging acquisition and processing

A single Biograph mMR 3 T PET/MRI scanner (Siemens Healthineers, Erlangen, Germany) with a 12-channel receiver array head coil was used to perform all MRI scans. Acquisition parameters were previously described.^23^ Briefly, 3 D T1-weighted and 3D FLAIR scans were acquired with 1.1 × 1.1 ×1.1 mm^3^ resolution, and segmented PCASL 3 D GraSE scans were acquired with the following parameters: 3.75 × 3.75 × 4 mm^3^ resolution, 36 axial slices, TE = 20.26 ms, TR = 4000 ms, labelling duration =1800 ms, post-labelling delay (PLD) = 1800 ms, 10 averages, 4 segments per volume, total scan time =320 s, background suppression, accompanied by an M0 image without labelling or background suppression (TR = 4000 ms).

The T1w and ASL images were processed using ExploreASL version 1.10.0.^30^ In short, grey matter (GM), white matter (WM), and CSF were segmented from T1-weighted images while correcting for WMH from FLAIR images. Bayesian Model Selection (BaMoS) was used to generate WMH segmentations and volumes from FLAIR and T1-weighted images.^31^ GM and deep WM regions-of-interest (ROIs) were created as an intersection of standard ROIs (from SPM12) with the individual CAT12 GM and WM segmentations (partial volume >0.5 in the ASL resolution) after subtraction of the WMH partial volume. The CAT12 WM ROI was eroded by a 4-voxel-sphere to form a deep WM ROI, before it was intersected with the subject-wise WM segmentation; this ROI is hereafter referred to as WM. ASL images were rigid-body registered to the T1w. The recommended single-compartment model was used to quantify CBF from the perfusion-weighted and M0 images in native space.^32^ Mean CBF (partial-volume corrected) and the ASL sCoV were calculated in GM and WM regions.^16^ All images were non-linearly registered to the Montreal Neurological Institute (MNI) standard space. The T1w, FLAIR, and ASL images were visually inspected by MD and HM (4 and 10 years of experience, respectively). Participants containing artefacts as described in Alsop et al. (2015),^33^ such as labelling, motion, or arterial transit artefacts, were excluded from all analyses. Exclusion and inclusion information is shown in Supplementary Figure 1.

### Statistical analysis

Statistical analyses were conducted in R version 4.2.1 (R Core Team, 2021). All data were tested for normal distribution using the Shapiro-Wilk test. GM sCoV, WM sCoV, and WMH were log-transformed because of their right-skewed distributions. To test if metrics differed between sexes per visit (cross-sectional analyses, Table 1), Independent Samples T-tests (normally distributed data) or Mann-Whitney-U tests (non-normally distributed data) were used. To test if cardiovascular risk factor metrics were correlated with age, differed between sexes longitudinally, and their interaction, we performed linear mixed models with a random intercept per subject (Table 2).

**Table 1.** Sample characteristics of life-course cardiovascular risk factors and imaging derivatives.

Age (years)	N	Overall	Males	Females
SBP				
36	254	118.1 ± 14.1***	122.7 ± 14.3	113.9 ± 12.5
43	269	121.8 ± 14.5***	125.1 ± 15.3	118.7 ± 13.0
53	276	133.2 ± 19.7***	141.1 ± 19.4	126.3 ± 17.2
60–64	282	135.9 ± 19.2***	142.1 ± 20.0	130.5 ± 16.7
69	276	135.4 ± 17.2***	139.0 ± 16.5	132.1 ± 17.2
69–71	281	139.7 ± 19.9***	144.3 ± 18.8	135.6 ± 20.2
DBP				
36	254	75.9 ± 11.5***	79.5 ± 11.4	72.8 ± 10.6
43	269	78.4 ± 11.1***	91.4 ± 11.1	75.7 ± 10.5
53	276	82.7 ± 12.2***	87.8 ± 12.2	78.3 ± 10.4
60–64	282	77.5 ± 10.1***	80.5 ± 10.3	74.7 ± 9.1
69	276	74.7 ± 9.9	75.4 ± 10.7	74.0 ± 9.3
69–71	281	81.5 ± 10.7	82.1 ± 13.3	81.1 ± 10.9
MAP				
36	254	90.1 ± 11.4***	93.9 ± 11.4	86.5 ± 10.2
43	269	92.9 ± 11.4***	95.9 ± 11.5	90.1 ± 10.5
53	276	99.6 ± 13.8***	105.6 ± 13.6	94.2 ± 11.8
60–64	282	96.9 ± 12.2***	101.0 ± 12.5	93.3 ± 10.7
69	276	94.8 ± 11.3*	96.6 ± 11.3	93.3 ± 11.0
69–71	281	100.9 ± 12.4*	102.8 ± 11.9	99.3 ± 12.7
PP				
36	254	42.1 ± 10.7	43.2 ± 10.9	41.1 ± 10.3
43	269	43.4 ± 10.2	43.8 ± 11.0	42.9 ± 9.5
53	276	50.5 ± 12.9***	53.4 ± 13.6	47.9 ± 11.8
60–64	282	48.5 ± 13.8**	61.5 ± 14.8	55.7 ± 12.2
69	276	60.7 ± 13.1***	63.6 ± 13.1	58.1 ± 12.5
69–71	281	58.2 ± 15.8***	62.3 ± 14.8	54.6 ± 15.8
FHS-CVS				
36	252	2.4 (1.4, 3.4)***	3.4 (2.7, 4.0)	1.45 (1.17, 1.9)
53	276	9.4 (5.4, 15)***	15 (11.8, 17.5)	5.55 (4.40, 7.88)
69	275	20.1 (13.1, 32.2)***	32.2 (25.7, 37.3)	13.3 (9.8, 16.6)
Imaging				
Total number of participants	282	282	133 (47.2%)	149 (52.8%)
Age at imaging (years)	282	70.6 ± 0.7	70.6 ± 0.7	70.7 ± 0.7
GM volume (mL)	282	592.4 ± 48.7***	620.3 ± 89.3	567.5 ± 38.7
WM volume (mL)	282	487.8 ± 58.3***	520.4 ± 52.1	458.6 ± 47.2
CSF volume (mL)	282	376.5 ± 66.3***	417.5 ± 55.5	339.9 ± 52.5
WMH volume (mL)	282	2.71 (0.8, 5.89)	2.32 (0.61, 5.06)	2.79 (0.92, 6.31)
GM CBF (mL/100g/min)	282	61.2 ± 14.8***	51.2 ± 13.1	67.4 ± 13.4
WM CBF (mL/100g/min)	282	22.3 ± 7.4***	18.7 ± 6.39	25.5 ± 6.76
GM sCoV (σ/μ)	282	0.46 ± 0.18***	0.47 ± 0.11	0.41 ± 0.27
WM sCoV (σ/μ)	282	0.71 ± 0.42***	0.75 ± 0.29	0.59 ± 0.27

Age is in years. Values shown are n (%), mean (SD), or median (q1, q3). The asterisk (*,**,***) denotes statistically significant differences between for male/female (p < 0.05, p < 0.01, p < 0.001, respectively). CBF: cerebral blood flow; DBP: diastolic blood pressure; FHS-CVS: Framingham Heart Study - Cardiovascular Risk Score; GM: grey matter; MAP: mean arterial pressure; PP: pulse pressure; SBP: systolic blood pressure; sCoV: spatial coefficient of variation; WM: white matter; WMH: white matter hyperintensity.

**Table 2.** Linear mixed effects models detailing the relation between age and each cardiovascular risk factor metric (SBP, DBP, MAP, PP, and FHS-CVS), including sex interactions.

	B	95% CI	p-value	Sex interaction (p-value)
SBP	0.58	0.51 – 0.66	<0.001	0.01 (0.70)
DBP	−0.06	−0.12 – −0.01	0.013	0.17 (<0.001)
MAP	0.15	0.10 – 0.20	<0.001	0.12 (0.0015)
PP	0.65	0.59 – 0.71	<0.001	−0.16 (<0.001)
FHS-CVS	0.86	0.82 – 0.89	<0.001	−0.5 (<0.001)

An asterisk indicates associations. B: unstandardised beta coefficient; CI: confidence interval; DBP: diastolic blood pressure; FHS-CVS: Framingham Heart Study - Cardiovascular Risk Score MAP: mean arterial pressure; PP: pulse pressure; SBP: systolic blood pressure.

To understand the effect of cardiovascular risk factors at each visit, linear regression models were performed to investigate the relationship between each ASL metric (GM CBF and log-transformed sCoV, and WM CBF and log-transformed sCoV) and life-course cardiovascular risk factor metrics (FHS-CVS at ages 36, 53 and 69 years; SBP, DBP, MAP and PP at ages 36, 43, 53, 60–64, 69 and 69–71 years). To understand the effect of changes in cardiovascular risk factors, we also calculated the between-visit differences in cardiovascular risk factors, further referred to as “the cardiovascular difference” metrics, and investigated the relationship of these differences with ASL metrics in linear regression models. Furthermore, linear regression models were performed to investigate the relationship between the ASL metrics and log-transformed WMH volume at age 69–71 years. All analyses were adjusted for age at scanning, and the cardiovascular difference analyses were additionally adjusted for the age differences within the compared visits. Because most of the metrics were statistically different between sexes (Table 1), sex was added as a covariate to all further analyses, and differential influences of sex were tested using an interaction term. The interaction effects are only shown if they were statistically significant (p < 0.05). To investigate the role of BP-lowering medication in the relationships between life-course cardiovascular risk factor metrics and ASL metrics, significant models were repeated with BP medication as covariate instead of correcting the BP values.

### Multiple comparison corrections

All statistical models were corrected for multiple comparisons using the Benjamini and Hochberg step-up procedure to maintain a false discovery rate (FDR) of 5%. All ASL metrics (GM CBF and sCoV, and WM CBF and sCoV) were considered as separate blocks. For each ASL metric, all BP measurements (SBP, DBP, MAP, PP, and FHS-CVS) at each time point (age 36, 43, 53, 60–64, 69, and 69–71 years) were considered as one block. WMH was not corrected for multiple testing, as only four comparisons were made (CBF and sCoV of both GM and WM).

## Results

For each visit, cardiovascular risk factors, brain volumes, and the sCoV were higher for males compared to females, and CBF was higher for females compared to males cross-sectionally (p < 0.05, Table 1). Only DBP at age 69 years (p = 0.2) and 69–71 years (p = 0.4), PP at age 36 (p = 0.1) and 43 years (p = 0.6), age at imaging visit (p = 0.201), and WMH volume (p = 0.4) did not differ between sex cross-sectionally (Table 1, Supplementary Figures 2–3). With all visits combined (Table 2), age was significantly associated with SBP (B = 0.58, p < 0.001), DBP (B = −0.06, p = 0.013), and MAP (B = 0.15, p < 0.001), PP (B = 0.65, p < 0.001), and FHS-CVS (B = 0.86, p < 0.001). Sex was significantly associated with SBP (p < 0.001), DBP (p < 0.001), MAP (p < 0.001), FHS-CVS (p < 0.001) but not with PP (p = 0.08, data not shown). The models with DBP (p < 0.001), MAP (p = 0.002) and PP (p < 0.001), and FHS-CVS (p < 0.001), except for SBP (p = 0.81), showed significant age-sex interactions.

### Life-course cardiovascular associations

**Figure 2.**
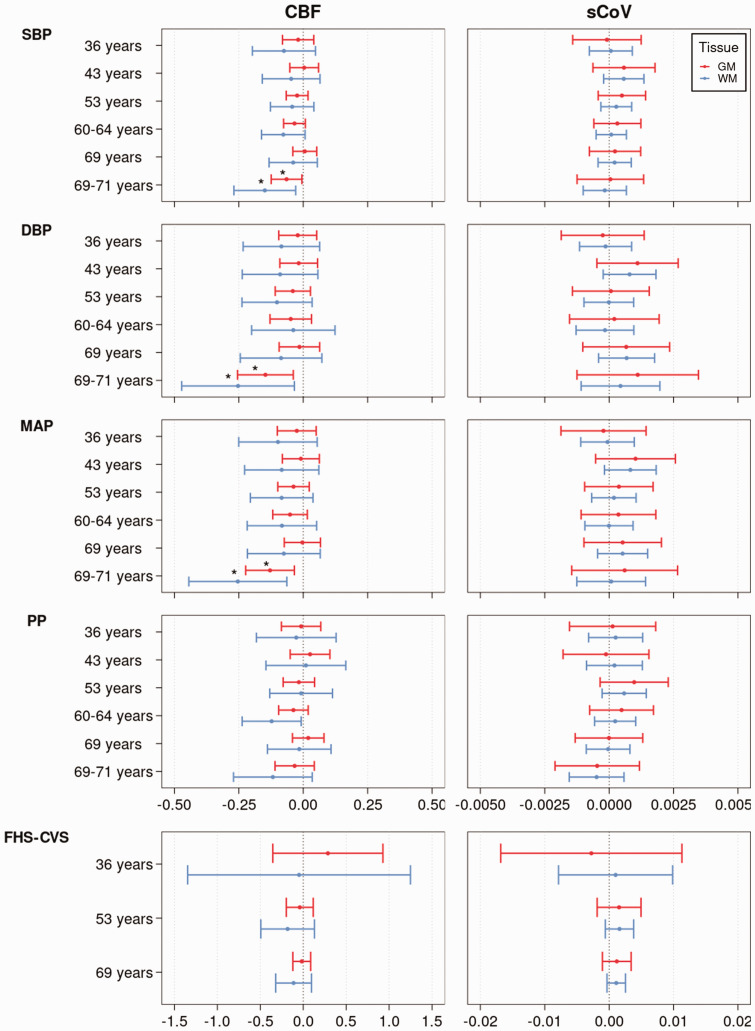
The relationship between CBF, sCoV, and life-course cardiovascular risk factor metrics (SBP, DBP, PP, MAP, FHS-CVS). Regression coefficient plot of the regression estimates that reflect the differences in mean CBF and sCoV, for GM (red) and WM (blue), change predicted by each cardiovascular risk factor metric corrected for sex and age at scan. The lines indicate the 95% confidence intervals. The asterisk indicates statistical significance after FDR correction. CBF: cerebral blood flow; DBP: diastolic blood pressure; FDR: false discovery rate; FHS-CVS: Framingham Heart Study – Cardiovascular Risk Score; GM: grey matter; MAP: mean arterial pressure; PP: pulse pressure; SBP: systolic blood pressure; sCoV: spatial Coefficient of Variation; WM: white matter.

After FDR correction, low GM CBF was associated with high SBP (B = −0.15, p = 0.029), including a sex interaction effect for this measure (male/female B = −0.15/0.02, p = 0.038), high DBP (B = −0.25, p = 0.031), and high MAP (B = −0.25, p = 0.029) measured at 69–71 years. Similarly, low WM CBF was associated with high SBP (B = −0.25, p = 0.031), DBP (B = −0.15, p = 0.016), and high MAP (B = −0.13, p = 0.016, Figure 2) measured at 69–71 years. Before FDR correction, low GM CBF was also associated with high PP before FDR correction (Supplementary Table 1) measured at 60–64 years. No significant associations (p > 0.05) were found between any of the other BP and FHS-CVS measurements and ASL metrics. No other sex interactions were found. Relationships between the ASL metrics and life-course cardiovascular measurements are plotted in Figure 2 and Supplementary Figures 3–8.

After FDR correction, low WM CBF was associated with increases in SBP (B = −0.07, p = 0.04), DBP (B = −0.13, p = 0.04), and MAP (B = −0.12, p = 0.04) measured between 69 to 69–71 years (Supplementary Table 2). Before FDR correction, associations were found between low GM CBF and increased PP between 53 and 60–64 years, low WM CBF and increased SBP between 60–69 years, and high GM sCOV and increased DBP and MAP between 36 and 43 years and (Supplementary Table 2). None of the other cardiovascular difference metrics were associated with ASL metrics (p > 0.05) before or after FDR corrections. No sex interactions were found. Regression results between the ASL metrics and (change in) life-course cardiovascular measurements before and after FDR corrections are shown in Supplementary Tables 1 and 2.

As a sensitivity analysis, use of BP-lowering medication at each age was added as a covariate instead of adjusting the BP values. Any BP measured at age 69–71, any BP change between 69 and 69–71 years, and BP-lowering medication were not associated with GM or WM CBF (p > 0.05, data not shown).

### WMH volume associations

**Figure 3.**
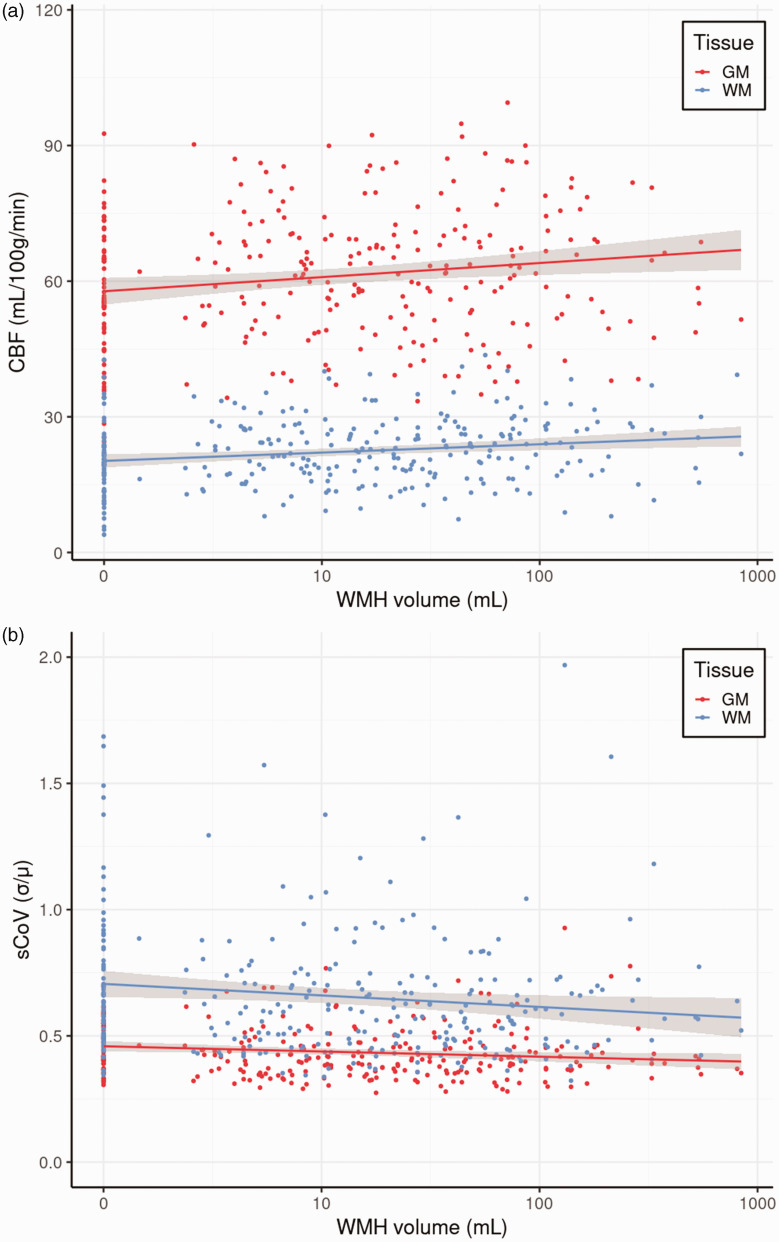
The relationships between WMH volume GM and WM CBF (a), and between WMH volume and GM and WM sCoV (b). CBF: cerebral blood flow; GM: grey matter; sCoV: spatial coefficient of variation; WM: white matter; WMH: white matter hyperintensity.

After FDR correction, high GM CBF (B = 4.56, p = 0.034, Figure 3(a)), high WM CBF (B = 3.08, p = 0.004, Figure 3(a)), low log-transformed GM sCoV (B = −0.03, p = 0.043, Figure 3(b)) and low log-transformed WM sCoV (B = −0.05, p = 0.037, Figure 3(b)), were associated with large log-transformed WMH volume, without sex interaction effects (p < 0.05).

### Post-hoc analyses

Further analyses investigating the statistically significant associations of SBP, DBP, and MAP with GM CBF measured at 69–71 years were performed by dividing the GM bilaterally into the anterior (ACA), middle (MCA), and posterior cerebral artery (PCA) vascular territories. Similar associations of SBP, DBP, and MAP were found with the ACA and MCA vascular territories after FDR correction (Supplementary Table 3, Supplementary Figure 9). No associations were found with the PCA vascular territory (Supplementary Table 3, Supplementary Figure 9).

The post-hoc analyses between cardiovascular risk factors and log-transformed WMH were performed similarly to the linear regression analyses between cardiovascular risk factors and ASL metrics, and revealed no associations between the cardiovascular risk factors at 69–71 years and log-transformed WMH (Supplementary Table 4) or for any of the other ages (p > 0.05, results not shown).

## Discussion

In this cognitively unimpaired cohort, we found that high late-life BP (measured at 69–71 years) and late-life increases in BP (measured between 69 and 69–71 years) were associated with low global late-life cerebral perfusion. In addition, high SBP was associated with low GM CBF for males but not for females. We did not find any associations of CBF or spatial CoV with any of the early- and mid-life cardiovascular risk factors. Secondly, both high CBF and low sCoV were associated with large WMH volume.

### Associations with cardiovascular health

Associations between BP and CBF varied greatly in previous studies, ranging from no association,^34^ a negative longitudinal association between MAP and CBF^35^ and between SBP and GM CBF,^36^ to no change^37^ or increases^37–39^ in GM CBF after anti-hypertensive treatment. Our late-life inverse associations of CBF with SBP and MAP are consistent with several cross-sectional studies,^36,40,41^ which could be explained by ageing-related changes such as vessel wall remodelling.^42^ In contrast, our inverse association between DBP and CBF seems less straightforward and was found only in two previous studies.^35,43^ This association may follow the same principle as our SBP association since MAP was also associated with CBF, but PP was not. Some other studies did not find any BP-CBF associations,^34,44^ perhaps because of their smaller sample sizes, different age distributions, BP averages and ranges, and imaging-related differences in PLD and analysis techniques resulting in large differences in measured GM CBF. Inclusion criteria may also play a role, as suggested by a recent longitudinal aging study which provided a post-hoc analysis, splitting their cohort into a sub-group with and without subjective cognitive impairment.^45^ A correlation between SBP and CBF was only found for the subgroup with subjective cognitive impairment in this study. Post-hoc analyses revealed similar associations of SBP, DBP, and MAP with the anterior circulation only. One explanation could be that the PCA's labelling efficiency is usually poorer and more heterogeneous because of the rather tortuous vertebral arteries.^46^ The absence of an association between BP and sCoV is in agreement with one previous study,^34^ suggesting that blood label arrival is not severely delayed and that vascular efficiency is not directly related to cardiovascular risk factors in normal ageing.

Previous Insight 46 studies found relationships of FHS-CVS with white matter integrity,^22^ WMHs,^29^ and biological brain age.^47^ Our absence of similar associations with ASL metrics is unexpected, especially as BP is an important component of FHS-CVS and it could be envisioned that relationships between systolic BP and CBF would contribute to relationships between FHS-CVS and CBF. Several other studies found relationships of CBF with FHS-CVS and Systematic COronary Risk Evaluation (SCORE).^48,49^ However, our results are in agreement with one other study that did not find any relationships between ASL metrics and vascular composite scores,^50^ although this study did not consider BP separately. The use of different vascular risk scores—such as different FHS-CVS versions—might explain the differences between all studies, including our study.^51,52^

In contrast with our results, previous Insight 46 studies found associations of early- and mid-life cardiovascular risk factors with late-life low brain volume, low white matter integrity, and high WMH volume.^21,22^ These structural metrics of long-term accumulated pathology are perhaps a more stable correlate with cardiovascular risk factors than a late-life global perfusion snapshot. Because CBF is a highly dynamic physiological biomarker, it is not only potentially sensitive to the earliest cerebrovascular changes but also to short-term physiological and cardiovascular fluctuations,^53^ this may reduce its discrimination as a biomarker for early small cerebrovascular changes. Our lack of associations of ASL with earlier cardiovascular risk factors could point to the fact that ASL typically measures time-average CBF—measured here across ∼5 min. Perhaps this time-average global CBF can be kept at a healthy level by cerebral autoregulation.^54^ However, it might still allow brief bouts of local hypo- or hyperperfusion that could lead to the abovementioned accumulated structural damage^55^ (although these may not necessarily be apparant during the ASL scan). Perhaps, localised cerebrovascular reactivity measurements, with or without cardiovascular stressors, would correlate better with structural damage than resting-state global CBF. To what extent our findings can be explained through hypoperfusion or other types of cerebral pathology, such as inflammation or blood-brain-barrier dysfunction, cannot be determined from our findings.

Our life-course cardiovascular risk factors were higher in males than females. Likewise, previous Insight 46 findings found that females cognitively outperform males and have a lower predicted biological brain age.^47,56^ Perhaps, this fits with our stronger statistical effects for males, even though most sex interactions were not statistically significant. The fact that we only found a significant sex interaction effect between SBP and CBF could be explained through vascular remodelling, which differs between men and women,^57,58^ and is more related to SBP than DBP.^59,60^ This sex-dependent vascular remodelling effect could be smaller for the WM as blood reaches the WM later than GM, dampening pulsatility effects,^61^ which could explain our absence of sex interaction effects between SBP and WM CBF. Another explanation might be that WM only requires minimal CBF to survive, in contrast with GM which requires BF to sustain changes in neuronal activity. This relatively low WM CBF leads to a low ASL WM signal-to-noise ratio,^62^ reducing the sensitivity of ASL for WM CBF effects; this could be another explanation for our absence of sex interaction effects between SBP and WM CBF.

The absence of sex interaction effects between SBP and WM CBF might be explained by the lower CBF requirement of WM, in contrast with GM which requires CBF to sustain neuronal activity. As the blood reaches WM later than the GM, and it consequently experiences lower pulsatility than GM, the effect of sex-dependent vascular remodelling could be smaller.^61^ Another explanation might be the relatively low sensitivity of ASL in the WM due to the low WM CBF requirements, resulting in a low ASL WM signal-to-noise ratio.^62^ Future studies should further explore the observed sex differences by expanding our analyses with regions at risk of WMH development, cerebrovascular reactivity, and including longitudinal CBF.^63,64^

### Associations with WMH volume

Most previous studies found associations of high WMH volume with low CBF and high sCoV in both healthy controls and patients,^19,65–67^ suggesting that hypoperfusion induces WMH development, potentially through ischemic tissue damage.^38,68,69^ Our results oppose these findings but are in agreement with another study that also found an association of large WMH volume with high CBF,^66^ and low sCoV, which could be interpreted as hyperperfusion as a compensatory effect on WMH.^66^

It is challenging to directly compare these studies with ours, as many studies investigating cerebral perfusion and WMH include patients with MCI or dementia.^69^ Indeed, the association between WMH and CBF weakened in one study after excluding MCI and or demented individuals.^70^ Individuals at risk of Alzheimer’s Disease (AD) demonstrate both increases and decreases in CBF, which is related to amyloid-beta pathology and brain atrophy.^15^ In addition to the typical neurovascular coupling—hypoperfusion reflecting decreased function and metabolism in neurodegenerating brain regions—hyperperfusion could reflect a compensatory effect in early disease stages to sustain a comparable level of functioning. This could suggest that the WMH-CBF associations differ between ageing-related WMH and AD-related WMH. Indeed, one study found differences in WMH histology between postmortem AD patients and controls.^71^ Future studies could investigate the role of CBF as a biomarker to differentiate between cardiovascular- and AD-related components of longitudinal WMH development.

### Post-hoc associations between cardiovascular risk factors and WMH volume

In contrast to Lane et al. (2019),^21^ who found associations between cardiovascular risk factors at 53 and WMH at 69 years, we did not find associations between life-course cardiovascular risk factors and WMH volume. This could reflect differences in the sample studied. We used a subset of the sample included by Lane et al. (2019), as 173 participants did not undergo an ASL scan or were excluded for the reasons shown in Figure 1 and mentioned in the methods and limitations. Our participants had a lower WMH load (n = 282, 4.4 ± 4.35 mL) than the participants without ASL data (n = 173, 6.2 ± 6.4 mL). Furthermore, compared to our participants, the participants without ASL data were more likely to be obese (40% vs 19%), more likely to have diabetes (17% vs 7%), and to be male (56% vs 47%). These differences could explain why our study could not reproduce the associations between cardiovascular risk factors and WMH in Lane et al. (2019), as the sample of Insight 46 participants who underwent ASL scanning and had usable CBF images were relatively healthy compared to the full sample used in Lane et al. (2019). Perhaps, global perfusion might be an earlier biomarker than WMH volume, as cardiovascular risk factors were already related to CBF but not with WMH loads in this relatively healthy Insight 46 subset.

### Limitations

The strength of our unique homogeneous and healthy cohort is, at the same time, a potential limitation. The Insight 46 participants appear to have relatively good cardiovascular health compared to the full NSHD cohort, perhaps due to a retention bias.^24,47^ Compared to reports of hypertension prevalence in the general population of 65% at age 65–74,^72^ the Insight 46 cohort only has a hypertension prevalence of 55% (at age 69–71 years).^21^ Additionally, participants with large vascular pathology such as infarcts were excluded from Insight 46. Moreover, ASL was the latest scan in the MRI protocol. If individuals experienced any claustrophobia or had trouble lying still, ASL would be the first scan to be excluded. Hence, the present ASL Insight 46 sample has a hypertension prevalence of 48% (at age 69–71 years), and therefore is healthier than the—already relatively healthy—Insight 46 cohort. It is thus possible that our findings are not representative for the general population, in which associations between early- or midlife cardiovascular risk factors and late-life cerebral haemodynamics could still exist. Another limitation of our relatively healthy cohort is that we cannot investigate the association of our findings with cognitive decline later in life. Although commonly used,^26,73–75^ the use of 10 mmHg (SBP) and 5 mmHg (DBP) corrections assume that all BP-lowering medicine users experience the same treatment effects, and effects of individual responses on the relationships between cardiovascular risk factors and cerebral haemodynamics can not be disentangled. Interestingly, when we corrected for antihypertensive medication as covariate instead of adjusting the BP measurement, the associations with CBF disappeared. Perhaps, the use of BP-lowering medication represents the prevention of long-term vascular damage, preserving cerebrovascular haemodynamics. Another potential limitation is the BP protocol change between the on-site Insight 46 visit (69–71 years, lying) and the NSHD at-home visits (all other visits, seated). BP measures in the supine position can differ from measures performed in a seated position.^76^ This could have biased the observed associations between differences in SBP, DBP, and MAP between 69 and 69–71 years and WM CBF. Furthermore, the on-site BP measurement could have induced a white coat effect,^77^ potentially explaining the average DBP increase between 69 years and 69–71 years (Figure 1). Our associations may have been further biased by the methodological constraints of ASL. Haematocrit measurements were not available for the CBF quantification, which may have especially biased our sex effects and sex interactions with age-dependent pathology.^78^ Females generally have lower levels of haematocrit than men, leading to both higher cerebral perfusion as a compensatory effect and higher blood T1 relaxation time, leading to an overestimation of CBF if not accounted for.^79^

## Conclusion

In conclusion, our study only revealed late-life associations between cardiovascular risk factors and cerebral hemodynamics. Furthermore, WMHs were associated with cerebral haemodynamics but not cardiovascular risk factors. These findings suggest that, in relatively healthy individuals, cerebral blood flow autoregulation keeps cerebral haemodynamics unaffected by cardiovascular risk factors until late-life. These findings encourage future life-span studies to investigate more subtle or sensitive ASL biomarkers, such as smaller regions or cerebrovascular reactivity measurements, to investigate the associations of cardiovascular risk factors with cerebral haemodynamics, WMH, and cognitive decline.

## Supplemental Material

sj-pdf-1-jcb-10.1177_0271678X241301261 - Supplemental material for Associations of life-course cardiovascular risk factors with late-life cerebral haemodynamics Supplemental material, sj-pdf-1-jcb-10.1177_0271678X241301261 for Associations of life-course cardiovascular risk factors with late-life cerebral haemodynamics by Mathijs BJ Dijsselhof, Jorina Holtrop, Sarah-Naomi James, Carole H Sudre, Kirsty Lu, Luigi Lorenzini, Lyduine E Collij, Catherine J Scott, Emily N Manning, David L Thomas, Marcus Richards, Alun D Hughes, David M Cash, Frederik Barkhof, Jonathan M Schott, Jan Petr and Henk JMM Mutsaerts in Journal of Cerebral Blood Flow & Metabolism
