# Supplementary material for: Associations of life-course cardiovascular risk factors with late-life cerebral hemodynamics
Source: J Cereb Blood Flow Metab. 2024 Nov 17;45(4):765–78. doi: 10.1177/0271678X241301261 (PMC11571377; doi:10.1177/0271678X241301261)
Supplement: sj-pdf-1-jcb-10.1177_0271678X241301261 - Supplemental material for Associations of life-course cardiovascular risk factors with late-life cerebral haemodynamics [file sj-pdf-1-jcb-10.1177_0271678X241301261.pdf]

1. Supplementary

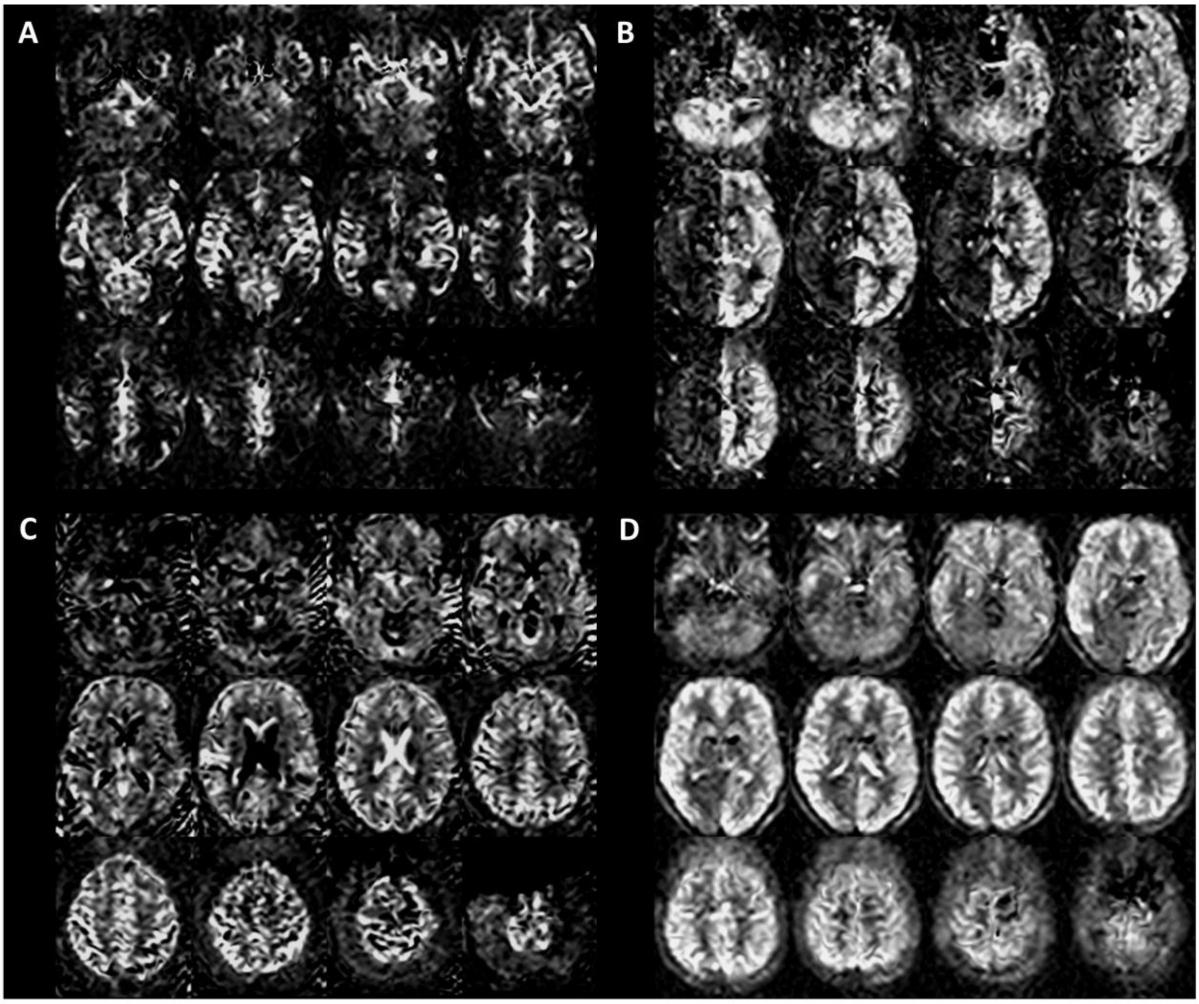

Supplementary Figure 1: Examples of excluded cerebral blood flow (CBF) images in Montreal Neurological Institute (MNI) standard space, showing arterial transit artefacts (A), asymmetrical labelling artefact (B), and motion artefacts (C). Example of an included representative CBF image (D), albeit with slight posterior labeling asymmetry. CBF = cerebral blood flow; MNI = Montreal Neurological Institute.

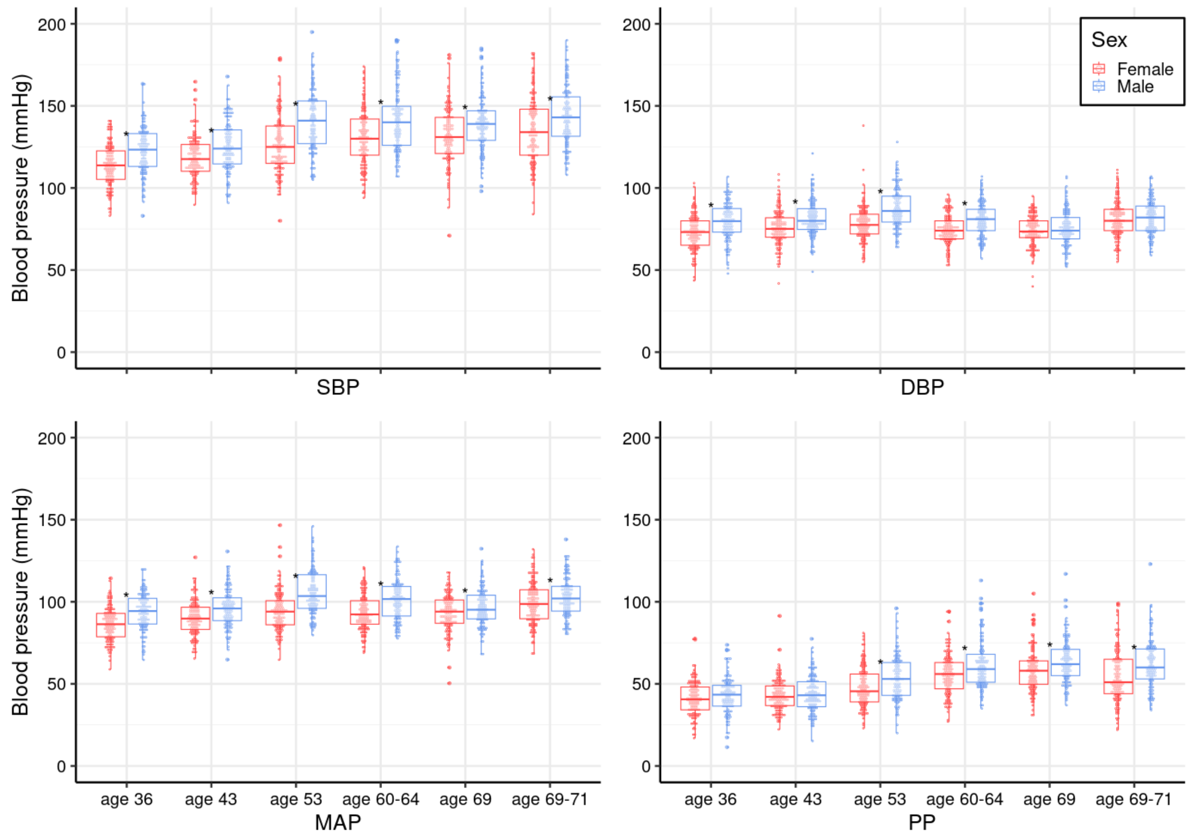

Supplementary Figure 2: Blood pressures at ages 36, 43, 53, 60-64, 69, and 69-71 years, stratified for sex. The asterisk denotes significant ($p < 0.05$) differences between male/female. DBP = diastolic blood pressure; MAP = mean arterial pressure; PP = pulse pressure; SBP = systolic blood pressure.

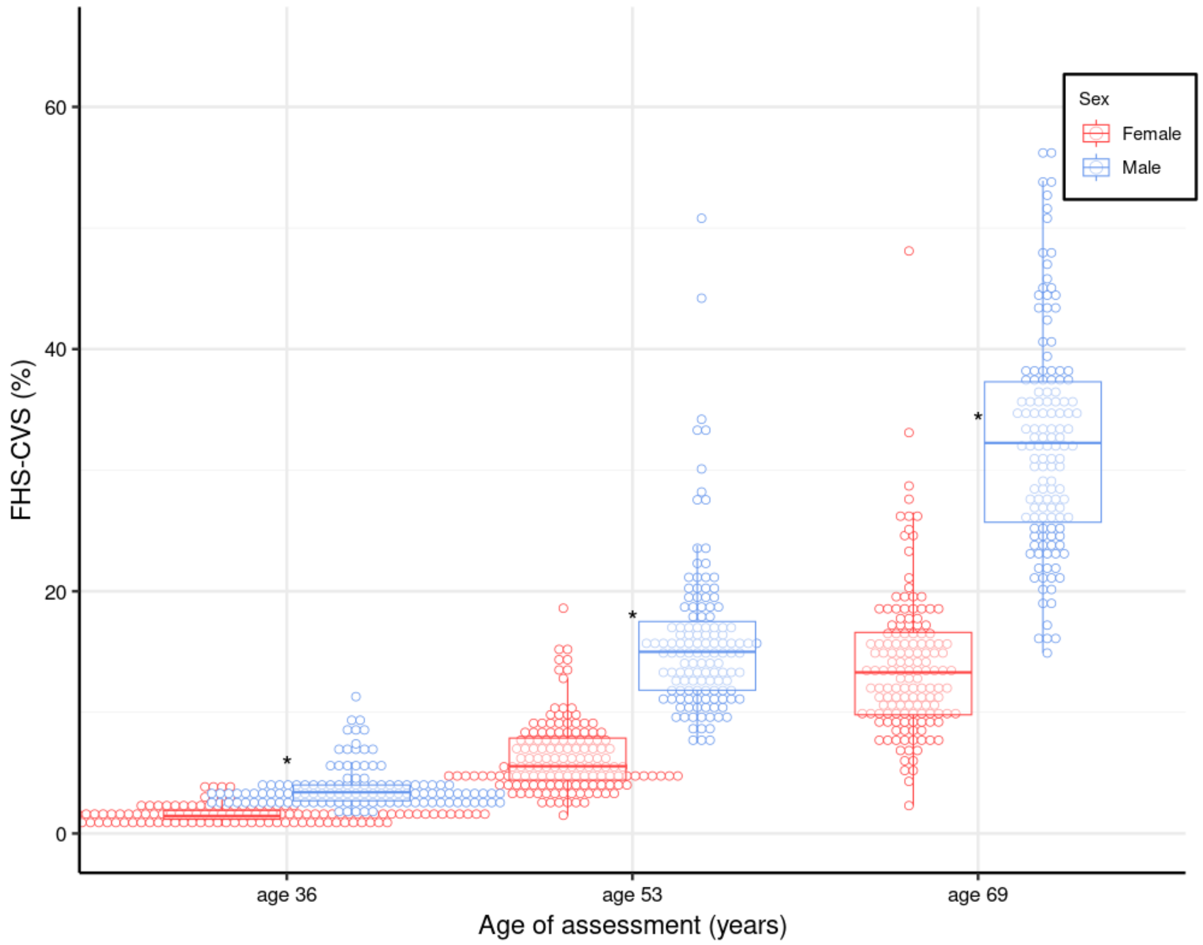

Supplementary Figure 3: FHS-CVS at ages 36, 53, and 69, stratified for sex. The asterisk denotes significant ($p < 0.05$) differences between male/female. FHS-CVS = Framingham Heart Study - Cardiovascular Risk Score.

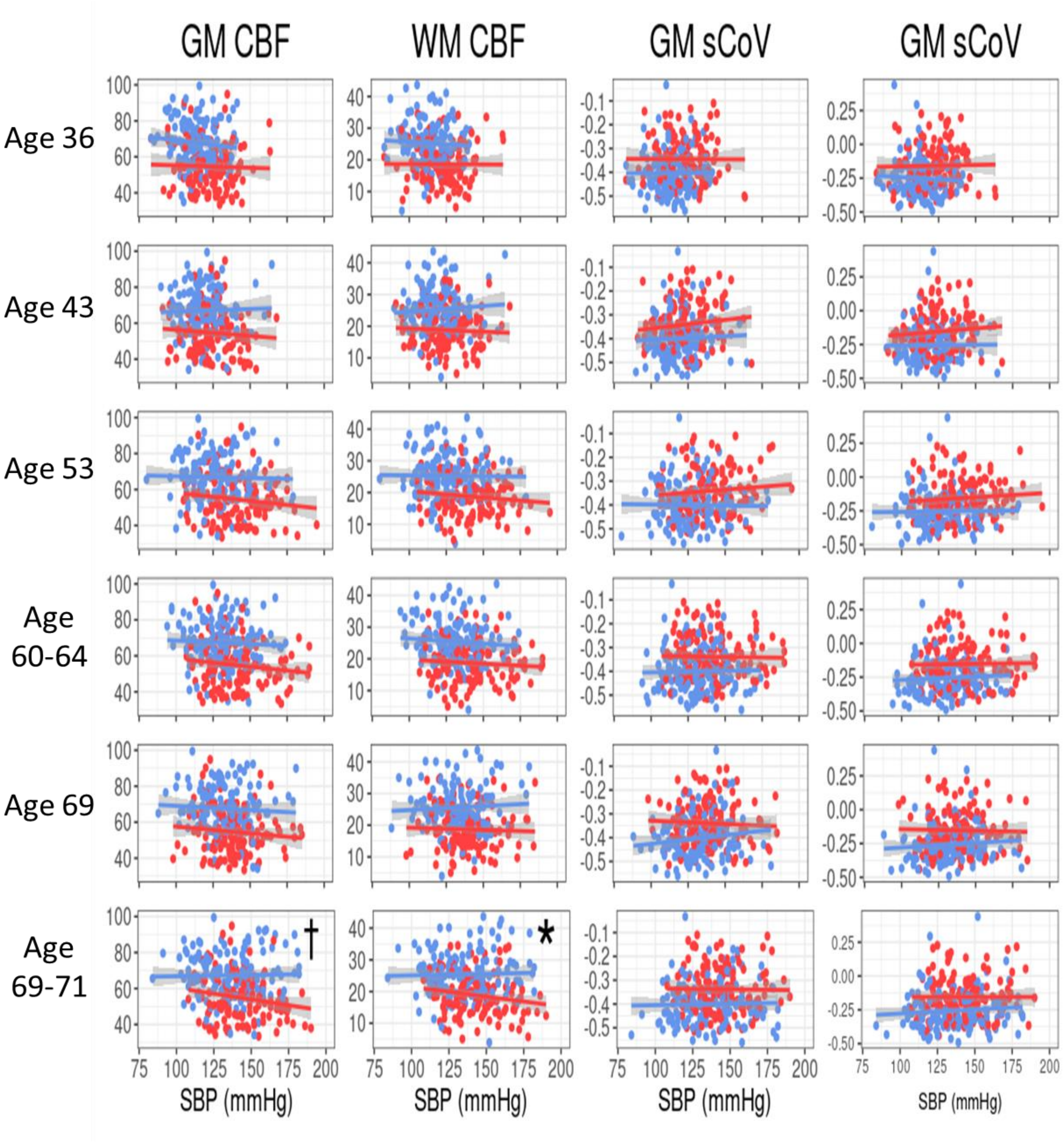

Supplementary Figure 4: The relationship between GM and WM CBF (mL/100g/min) and GM and WM sCoV (log-transformed) and life-course SBP, corrected for sex and age at scan. Males are shown in blue and females in red for visualisation purposes. The asterisk denotes FDR-corrected statistically significant associations for the whole sample, the cross sign denotes a significant sex-interaction effect. Abbreviations: CBF = cerebral blood flow; FDR = false discovery rate; GM = grey matter; SBP = systolic blood pressure; sCoV = spatial coefficient of variation; WM = white matter.

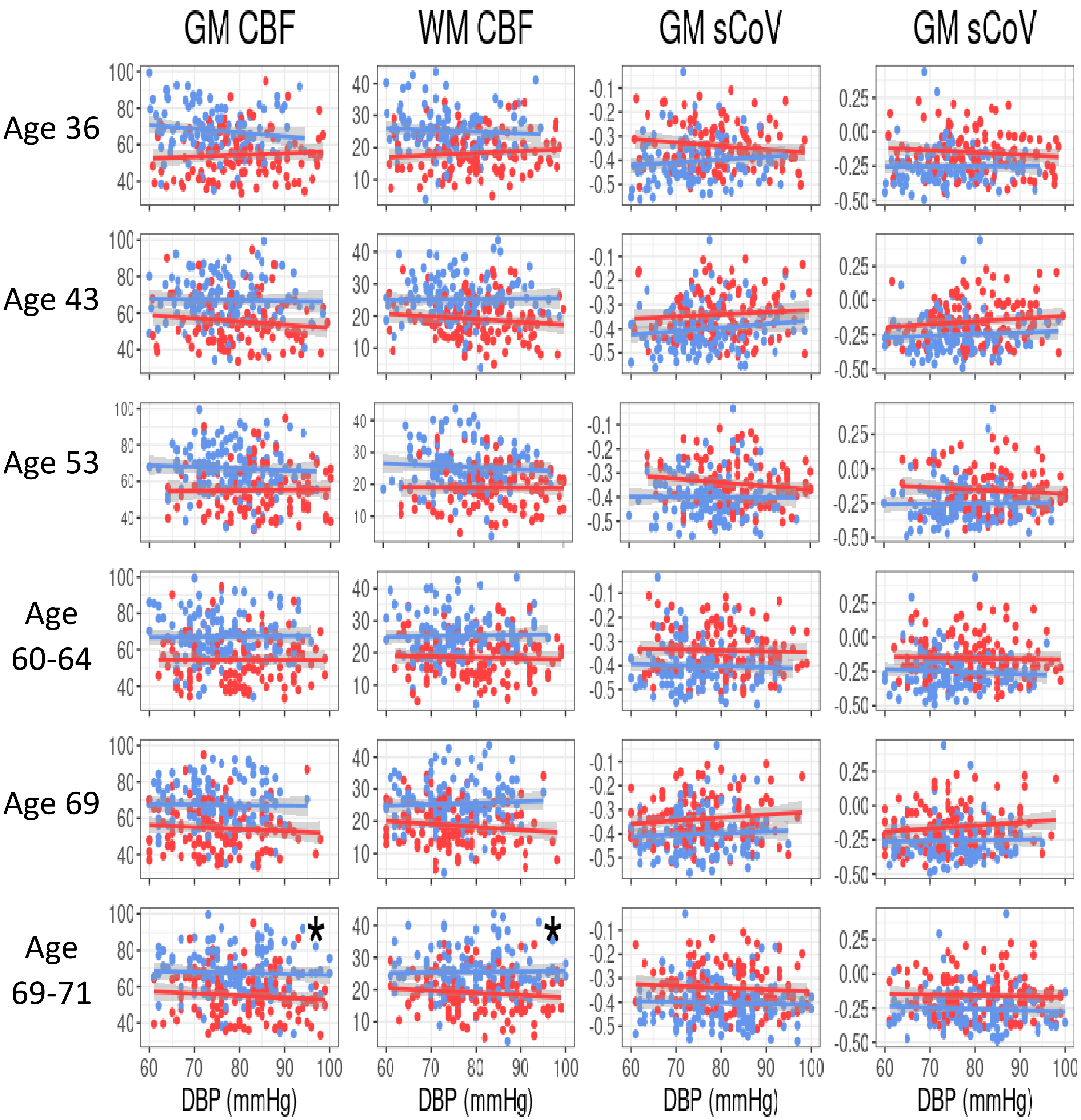

Supplementary Figure 5: The relationship between GM and WM CBF (mL/100g/min) and GM and WM sCoV (log-transformed) and life-course DBP, corrected for sex and age at scan. Males are shown in blue and females in red for visualisation purposes. The asterisk denotes FDR-corrected statistically significant associations. CBF = cerebral blood flow; DBP = diastolic blood pressure; FDR = false discovery rate; GM = grey matter; sCoV = spatial coefficient of variation; WM = white matter.

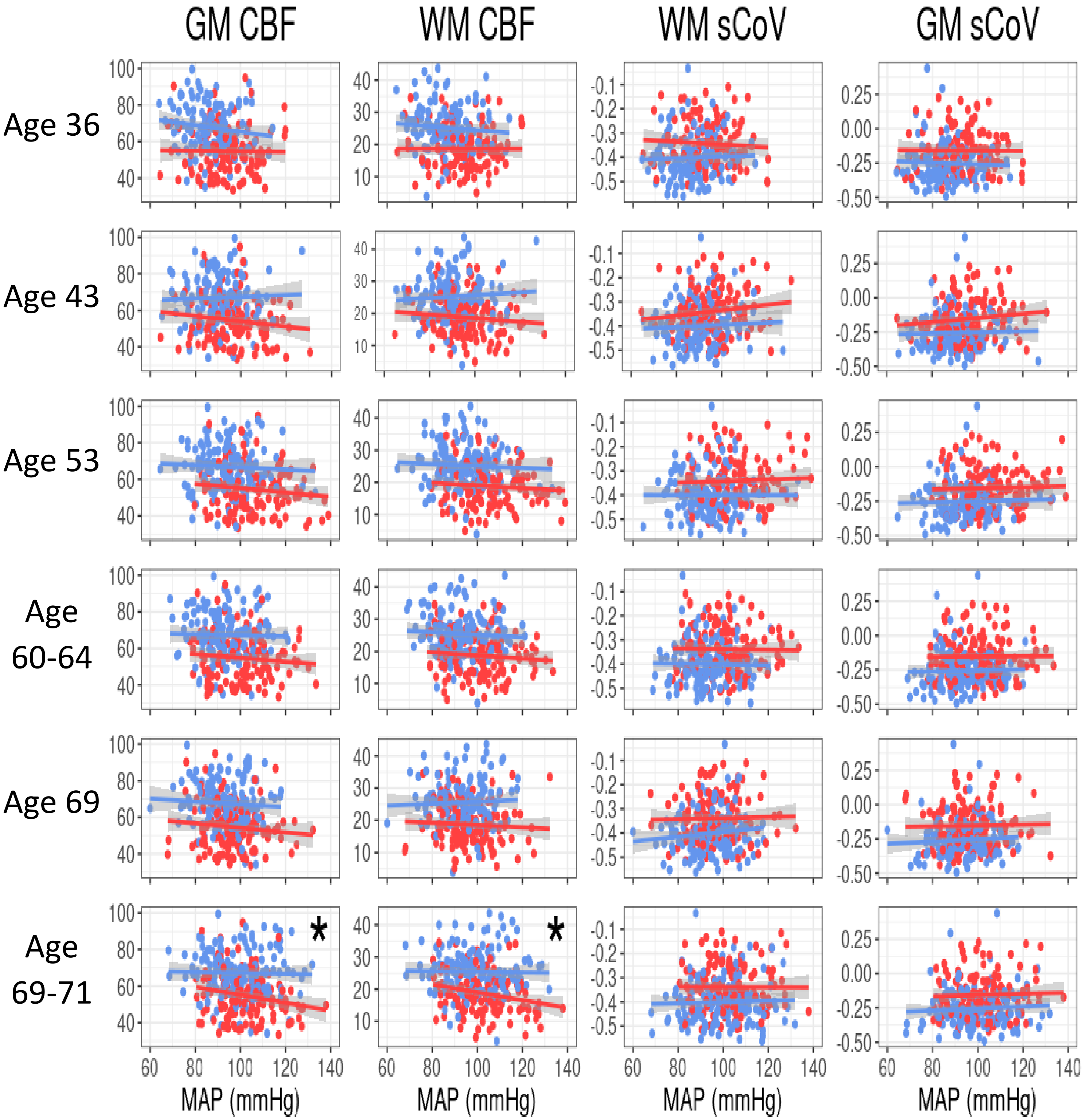

Supplementary Figure 6: The relationship between GM and WM CBF (mL/100g/min) and GM and WM sCoV (log-transformed) and life-course MAP, corrected for sex and age at scan. Males are shown in blue and females in red for visualisation purposes. The asterisk denotes FDR-corrected statistically significant associations. CBF = cerebral blood flow; FDR = false discovery rate; GM = grey matter; MAP = mean arterial pressure; sCoV = spatial coefficient of variation; WM = white matter.

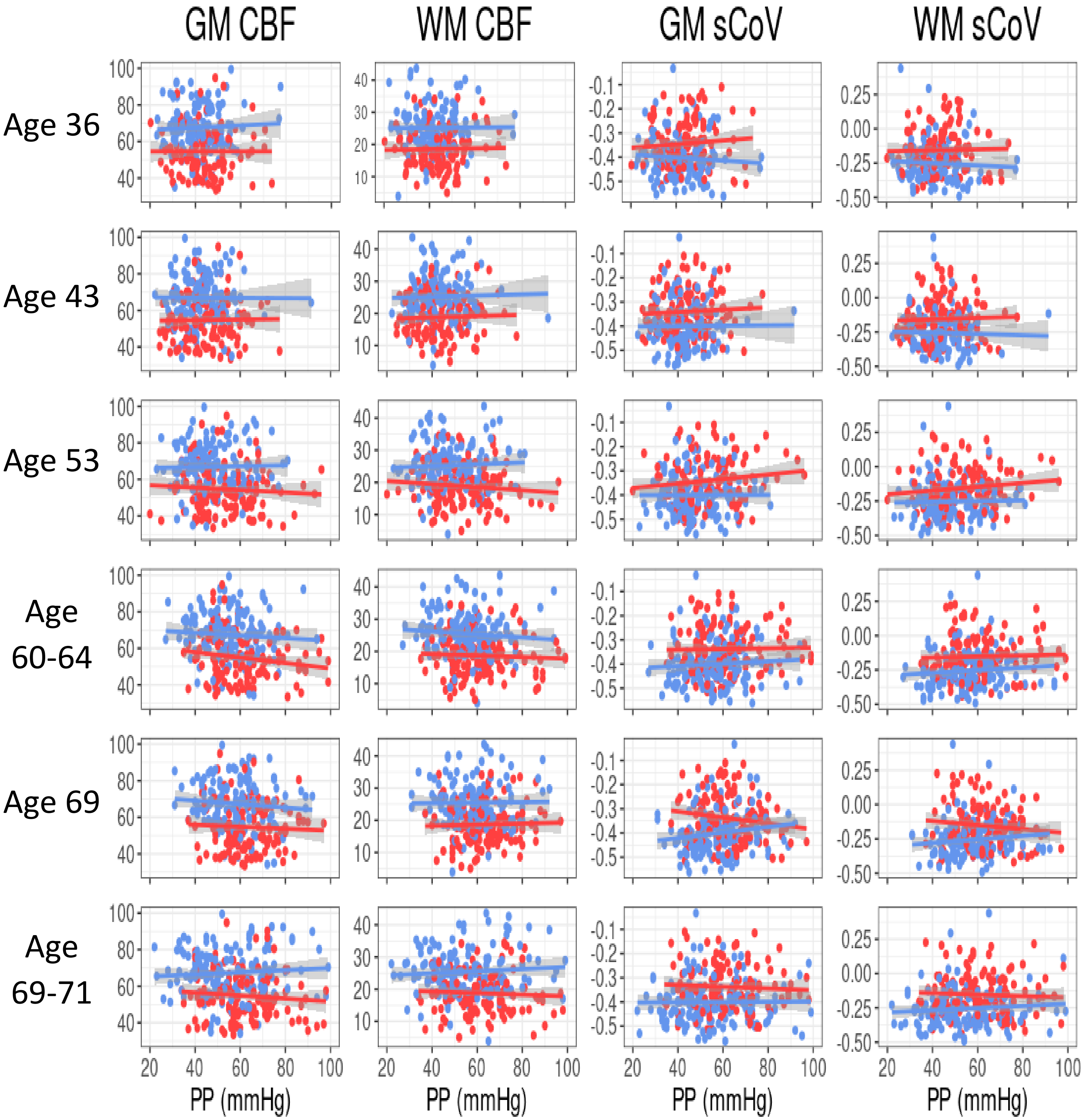

Supplementary Figure 7: The relationship between GM and WM CBF (mL/100g/min) and GM and WM sCoV (log-transformed) and life-course PP, corrected for sex and age at scan. Males are shown in blue and females in red for visualisation purposes. No significant associations ($p > 0.05$) were found. CBF = cerebral blood flow; GM = grey matter; PP = pulse pressure; sCoV = spatial coefficient of variation; WM = white matter.

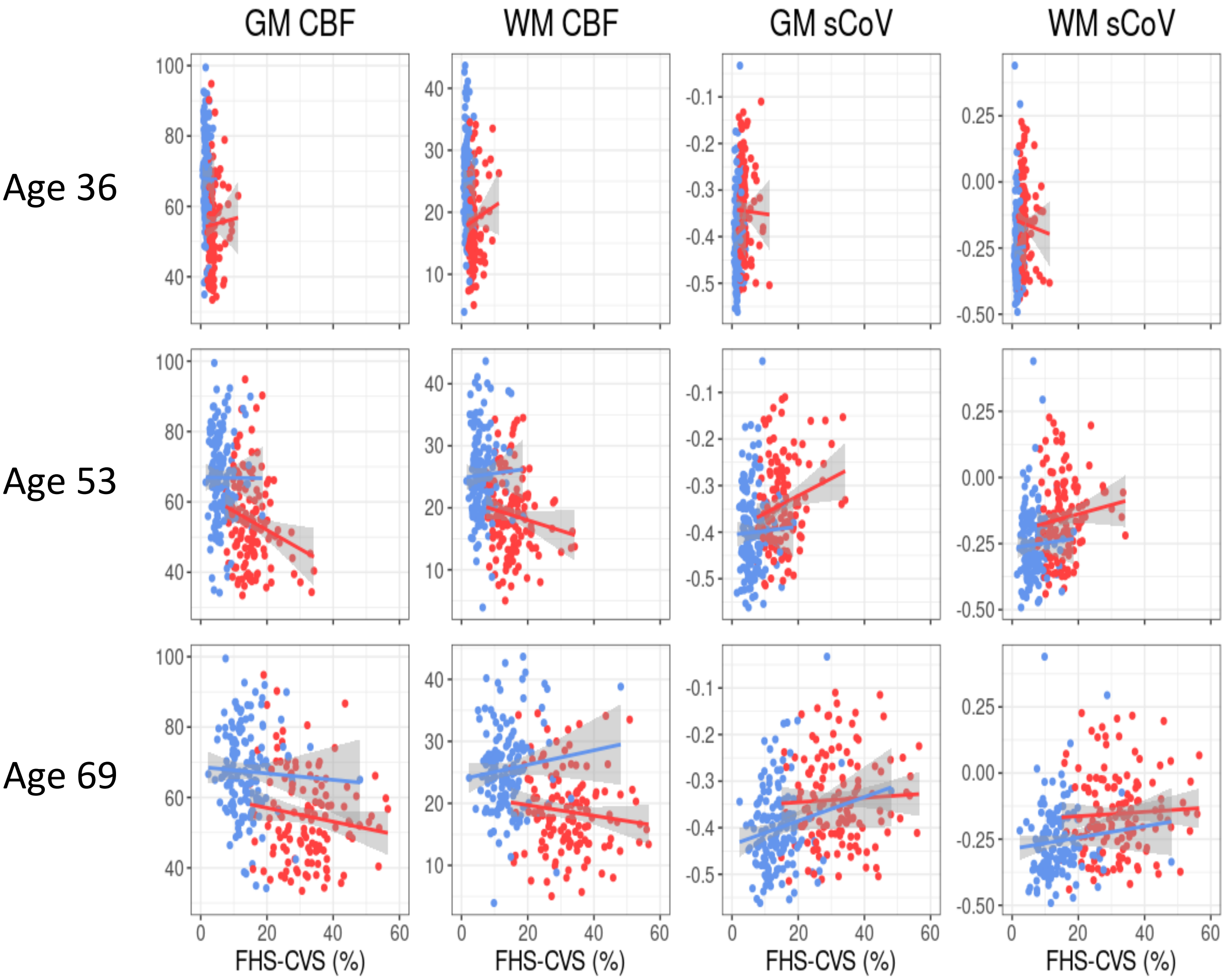

Supplementary Figure 8: The relationship between GM and WM CBF (mL/100g/min) and GM and WM sCoV (log-transformed) and life-course FHS-CVS, corrected for sex and age at scan. Males are shown in blue and females in red for visualisation purposes. No significant associations ($p > 0.05$) were found. CBF = cerebral blood flow; WM = white matter, FHS-CVS = Framingham Heart study - Cardiovascular Risk Score; sCoV = spatial coefficient of variation.

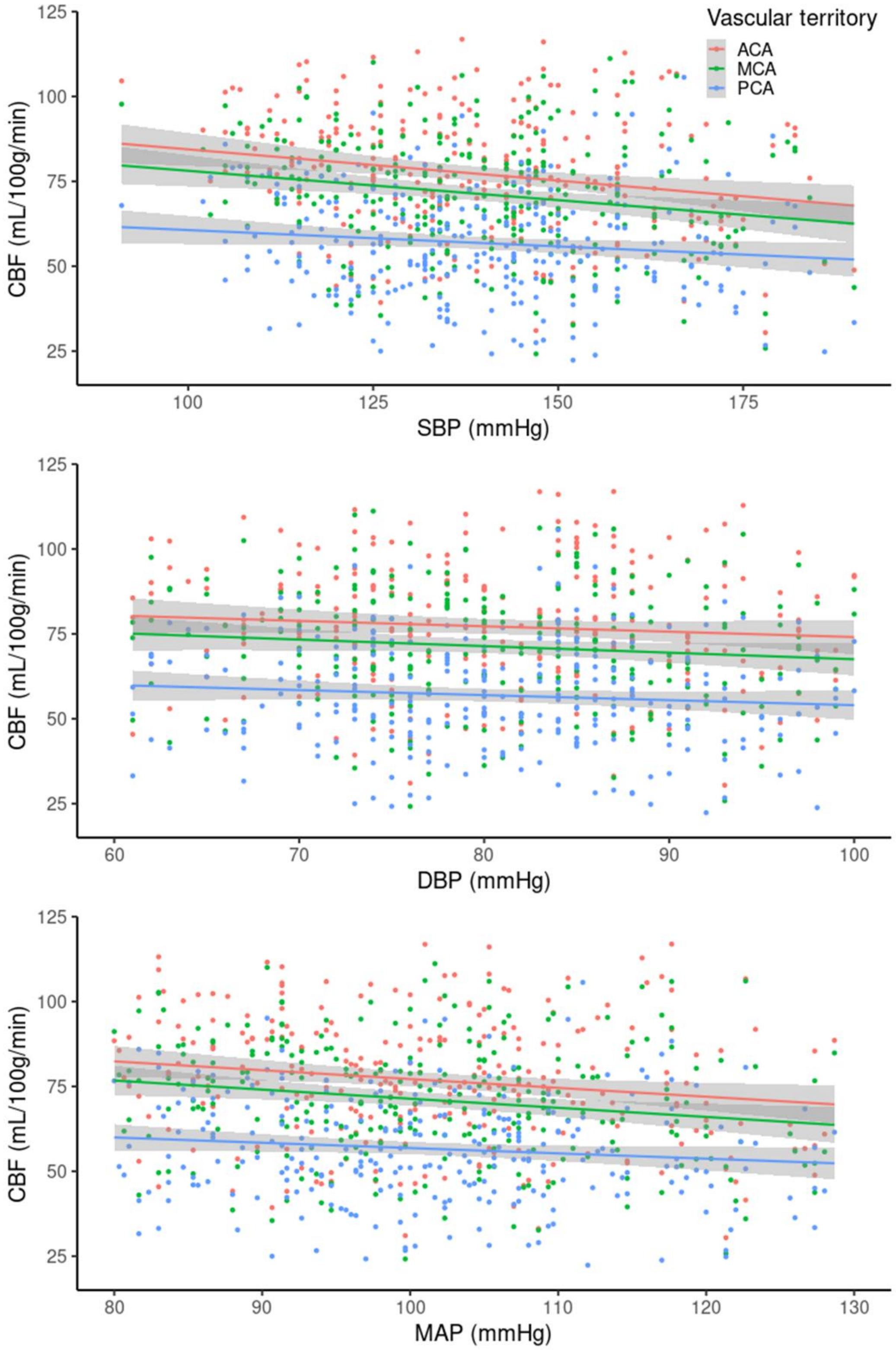

Supplementary Figure 9: The relationship between ACA (red), MCA (green), and PCA (blue) CBF (mL/100g/min), and SBP, DBP, and MAP at 69-71 years, corrected for sex and age at scan. ACA = anterior cerebral artery, CBF = cerebral blood flow; MCA = middle cerebral artery; PCA = posterior cerebral artery.

Supplementary Table 1: Linear regression models between GM CBF and WM CBF, and GM sCoV and WM sCoV, and each cardiovascular risk factor metric (SBP, DBP, MAP, PP, and FHS-CVS). All metrics were adjusted for sex and age at scan (and for sex-interaction effects if statistically significant). An asterisk indicates associations that survived FDR correction. B = unstandardised beta; CBF = cerebral blood flow; DBP = diastolic blood pressure; FDR = false discovery rate; FHS-CVS = Framingham Heart study - Cardiovascular Risk Score; GM = grey matter; MAP = mean arterial pressure; PP = pulse pressure; SBP = systolic blood pressure; sCoV = spatial coefficient of variation; WM = white matter.

Age [years]	GM CBF B *[95% CI]* (p-value, adjusted p-value)	WM CBF B *[95% CI]* (p-value, adjusted p-value)	GM sCoV B *[95% CI]* (p-value, adjusted p-value)	WM sCoV B *[95% CI]* (p-value, adjusted p-value)
SBP				
36	-0.07 *[-0.20 – 0.05]* (0.23, 0.44)	-0.02 *[-0.08 – 0.04]* (0.52, 0.71)	0.00 *[0.00 – 0.00]* (0.82, 0.90)	0.00 *[0.00 – 0.00]* (0.90, 0.90)
43	-0.05 *[-0.16 – 0.07]* (0.41, 0.55)	0.00 *[-0.05 – 0.06]* (0.90, 0.90)	0.00 *[0.00 – 0.00]* (0.15, 0.21)	0.00 *[0.00 – 0.00]* (0.35, 0.47)
53	-0.04 *[-0.13 – 0.04]* (0.32, 0.40)	-0.02 *[-0.07 – 0.02]* (0.27, 0.45)	0.00 *[0.00 – 0.00]* (0.36, 0.60)	0.00 *[0.00 – 0.00]* (0.29, 0.62)
60-64	-0.08 *[-0.16 – 0.01]* (0.07, 0.15)	-0.03 *[-0.08 – 0.01]* (0.11, 0.24)	0.00 *[0.00 – 0.00]* (0.79, 0.99)	0.00 *[0.00 – 0.00]* (0.49, 0.82)
69	-0.04 *[-0.13 – 0.05]* (0.41, 0.52)	-0.01 *[-0.04 – 0.05]* (0.80, 0.92)	0.00 *[0.00 – 0.00]* (0.52, 0.86)	0.00 *[0.00 – 0.00]* (0.71, 0.89)
69-71	-0.15 *[-0.27 – -0.03]* (0.02, 0.03)*	-0.06 *[-0.12 – -0.01]* (0.03, 0.04)*	0.00 *[0.00 – 0.00]* (0.69, 0.91)	0.00 *[0.00 – 0.00]* (0.94, 0.94)
69-71 Male	-0.15 *[-0.27 – -0.03]* (0.02, 0.03)*			
69-71 Female	0.02 *[-0.08 – 0.13]* (0.71, 0.71)			
DBP				
36	-0.08 *[-0.23 – 0.06]* (0.262, 0.436)	-0.02 *[-0.09 – 0.05]* (0.568, 0.712)	0.00 *[0.00 – 0.00]* (0.787, 0.903)	0.00 *[0.00 – 0.00]* (0.760, 0.899)
43	-0.09 *[-0.24 – 0.06]* (0.229, 0.510)	-0.02 *[-0.09 – 0.06]* (0.637, 0.898)	0.00 *[0.00 – 0.00]* (0.129, 0.205)	0.00 *[0.00 – 0.00]* (0.171, 0.389)
53	-0.10 *[-0.24 – 0.03]* (0.142, 0.396) [	-0.04 *[-0.11 – 0.03]* (0.247, 0.453)	0.00 *[0.00 – 0.00]* (0.979, 0.979)	0.00 *[0.00 – 0.00]* (0.931, 0.931)
60-64	-0.04 *[-0.20 – 0.12]* (0.640, 0.639)	-0.05 *[-0.13 – 0.03]* (0.237, 0.237)	0.00 *[0.00 – 0.00]* (0.755, 0.988)	0.00 *[0.00 – 0.00]* (0.819, 0.819)
69	-0.09 *[-0.24 – 0.07]* (0.285, 0.490) [	-0.01 *[-0.09 – 0.06]* (0.709, 0.923)	0.00 *[0.00 – 0.00]* (0.347, 0.859)	0.00 *[0.00 – 0.00]* (0.504, 0.839)
69-71	-0.25 *[-0.47 – -0.03]* (0.023, 0.035)*	-0.15 *[-0.26 – -0.04]* (0.008, 0.016)*	0.00 *[0.00 – 0.00]* (0.571, 0.905)	0.00 *[0.00 – 0.00]* (0.355, 0.770)
MAP				
36	-0.10 *[-0.25 – 0.05]* (0.206, 0.436)	-0.03 *[-0.10 – 0.05]* (0.512, 0.712)	0.00 *[0.00 – 0.00]* (0.903, 0.903)	0.00 *[0.00 – 0.00]* (0.793, 0.899)
43	-0.08 *[-0.23 – 0.06]* (0.255, 0.510)	-0.01 *[-0.08 – 0.06]* (0.800, 0.898)	0.00 *[0.00 – 0.00]* (0.108, 0.205)	0.00 *[0.00 – 0.00]* (0.194, 0.389)
53	0.08 *[-0.21 – 0.04]* (0.176, 0.396)	-0.04 *[-0.10 – 0.02]* (0.223, 0.453)	0.00 *[0.00 – 0.00]* (0.673, 0.842)	0.00 *[0.00 – 0.00]* (0.577, 0.721)
60-64	-0.08 *[-0.22 – 0.05]* (0.227, 0.302)	-0.05 *[-0.12 – 0.02]* (0.135, 0.237)	0.00 *[0.00 – 0.00]* (0.988, 0.988)	0.00 *[0.00 – 0.00]* (0.625, 0.819)
69	-0.08 *[-0.22 – 0.07]* (0.295, 0.490)	-0.00 *[-0.07 – 0.07]* (0.924, 0.923)	0.00 *[0.00 – 0.00]* (0.783, 0.925)	0.00 *[0.00 – 0.00]* (0.492, 0.839)
69-71	-0.25 *[-0.44 – -0.06]* (0.009, 0.029)*	-0.13 *[-0.22 – -0.03]* (0.008, 0.016)*	0.00 *[0.00 – 0.00]* (0.905, 0.905)	0.00 *[0.00 – 0.00]* (0.565, 0.770)

PP				
36	0.03 *[-0.18 – 0.13]* (0.730, 0.913)	-0.01 *[-0.08 – 0.07]* (0.831, 0.831)	0.00 *[0.00 – 0.00]* (0.637, 0.903)	0.00 *[0.00 – 0.00]* (0.875, 0.899)
43	0.01 *[-0.14 – 0.17]* (0.898, 0.898)	0.03 *[-0.05 – 0.10]* (0.500, 0.898)	0.00 *[0.00 – 0.00]* (0.710, 0.710)	0.00 *[0.00 – 0.00]* (0.884, 0.884)
53	-0.01 *[-0.13 – 0.11]* (0.895, 0.895)	-0.02 *[-0.08 – 0.04]* (0.584, 0.614)	0.00 *[0.00 – 0.00]* (0.182, 0.389)	0.00 *[0.00 – 0.00]* (0.149, 0.622)
60-64	-0.12 *[-0.24 – -0.01]* (0.036, 0.143)	-0.04 *[-0.10 – 0.02]* (0.193, 0.237)	0.00 *[0.00 – 0.00]* [0.567, 0.988)	0.00 *[0.00 – 0.00]* (0.444, 0.819)
69	-0.02 *[-0.14 – 0.11]* (0.804, 0.804)	0.02 *[-0.04 – 0.08]* (0.534, 0.923)	0.00 *[0.00 – 0.00]* (0.925, 0.925)	0.00 *[0.00 – 0.00]* (0.992, 0.992)
69-71	0.12 *[-0.27 – 0.03]* (0.130, 0.130)	-0.03 *[-0.11 – 0.04]* (0.390, 0.390)	0.00 *[0.00 – 0.00]* (0.369, 0.905)	0.00 *[0.00 – 0.00]* (0.578, 0.770)
FHS-CVS				
36	-0.05 *[-1.35 – 1.25]* (0.941, 0.931)	0.29 *[-0.35 – 0.93]* (0.379, 0.711)	0.00 *[0.00 – 0.00]* (0.824, 0.903)	0.00 *[0.00 – 0.00]* (0.699, 0.899)
53	-0.18 *[-0.49 – 0.13]* (0.253, 0.396)	-0.04 *[-0.20 – -0.12]* (0.614, 0.614)	0.00 *[0.00 – 0.00]* (0.152, 0.389)	0.00 *[0.00 – 0.00]* (0.373, 0.622)
69	-0.11 *[-0.32 – 0.10]* (0.289, 0.490)	-0.02 *[-0.12 – 0.09]* (0.740, 0.923)	0.00 *[0.00 – 0.00]* (0.131, 0.925)	0.00 *[0.00 – 0.00]* (0.297, 0.839)

Supplementary Table 2: Linear regression models between ASL metrics (GM CBF and sCoV, WM CBF and sCoV) and change in cardiovascular risk factor metric (SBP, DBP, MAP, PP, and FHS-CVS) between each visit (age 36, 43, 53, 60-64, 69 and 69-71 years), adjusted for sex, age at scan and time difference between visits. An asterisk indicates associations that survived FDR correction. The asterisk denotes a significant relationship between predictor and outcome measure. CBF = cerebral blood flow; DBP = diastolic blood pressure; FDR = false discovery rate; FHS-CVS = Framingham Heart study - Cardiovascular Risk Score; GM = grey matter; MAP = mean arterial pressure; PP = pulse pressure; SBP = systolic blood pressure; sCoV = spatial coefficient of variation; WM = white matter.

Age [years]	GM CBF B *[95% CI]* (p-value, adjusted p-value)	WM CBF B *[95% CI]* (p-value, adjusted p-value)	GM sCoV B *[95% CI]* (p-value, adjusted p-value)	WM sCoV B *[95% CI]* (p-value, adjusted p-value)
SBP				
36 to 43	0.03 [-0.08 – 0.15] (0.592, 0.789)	0.02 [-0.04 – 0.08] (0.495, 0.785)	0.00 [-0.00 – 0.00] (0.234, 0.312)	0.00 [-0.00 – 0.00] (0.315, 0.420)
43 to 53	0.00 [-0.09 – 0.09] (0.988, 0.988)	-0.01 [-0.06 – 0.03] (0.522, 0.842)	-0.00 [-0.00 – 0.00] (0.702, 0.702)	0.00 [-0.00 – 0.00] (0.946, 0.946)
53 to 60-64	-0.04 [-0.13 – 0.05] (0.356, 0.475)	-0.01 [-0.06 – 0.03] (0.567, 0.893)	-0.00 [-0.00 – 0.00] (0.635, 0.853)	-0.00 [-0.00 – 0.00] (0.709, 0.965)
60-64 to 69	0.05 [-0.04 – 0.14] (0.283, 0.580)	0.05 [0.00 – 0.09] (0.046, 0.101)	0.00 [-0.00 – 0.00] (0.687, 0.681)	-0.00 [-0.00 – 0.00] (0.748, 0.900)
69 to 69-71	-0.03 [-0.12 – 0.06] (0.571, 0.740)	-0.07 [-0.14 – -0.01] (0.028, 0.037)*	-0.00 [-0.00 – 0.00] (0.528, 0.681)	0.00 [-0.00 – 0.00] (0.565, 0.770)
DBP				
36 to 43	-0.01 [-0.15 – 0.14] (0.897, 0.897)	0.01 [-0.06 – 0.08] (0.803, 0.803)	0.00 *[0.00 – 0.00]* (0.023, 0.056)	0.00 [-0.00 – 0.00] (0.113, 0.246)
43 to 53	0.00 [-0.13 – 0.14] (0.965, 0.988)	-0.01 [-0.07 – 0.06] (0.842, 0.842)	-0.00 [-0.00 – 0.00] (0.096, 0.385)	-0.00 [-0.00 – 0.00] (0.160, 0.370)
53 to 60-64	0.07 [-0.06 – 0.20] (0.304, 0.475)	0.01 [-0.06 – 0.07] (0.868, 0.893)	-0.00 [-0.00 – 0.00] (0.863, 0.853)	-0.00 [-0.00 – 0.00] (0.995, 0.965)
60-64 to 69	-0.05 [-0.20 – 0.11] (0.577, 0.740)	0.03 [-0.04 – 0.11] (0.357, 0.392)	0.00 [-0.00 – 0.00] (0.142, 0.551)	0.00 [-0.00 – 0.00] (0.657, 0.900)
69 to 69-71	-0.10 [-0.26 – 0.06] (0.225, 0.585)	-0.13 [-0.24 – -0.01] (0.028, 0.037)*	-0.00 [-0.00 – 0.00] (0.598, 0.681)	0.00 [-0.00 – 0.00] (0.818, 0.770)
MAP				
36 to 43	0.17 [-0.40 – 0.06] (0.148, 0.591)	-0.07 [-0.19 – 0.04] (0.207, 0.785)	0.00 *[0.00 – 0.00]* (0.028, 0.056)	0.00 [-0.00 – 0.00] (0.123, 0.246)
43 to 53	0.00 [-0.13 – 0.13] (0.971, 0.988)	-0.01 [-0.08 – 0.05] (0.660, 0.842)	-0.00 [-0.00 – 0.00] (0.208, 0.415)	-0.00 [-0.00 – 0.00] (0.383 0.510)
53 to 60-64	0.01 [-0.11 – 0.14] (0.824, 0.814)	0.00 [-0.07 – 0.06] (0.873, 0.893)	-0.00 [-0.00 – 0.00] (0.737, 0.853)	-0.00 [-0.00 – 0.00] (0.854, 0.965)
60-64 to 69	0.01 [-0.13 – 0.15] (0.833, 0.856)	0.05 [-0.02 – 0.12] (0.122, 0.182)	0.00 [-0.00 – 0.00] (0.285, 0.558)	0.00 [-0.00 – 0.00] (0.900, 0.900)
69 to 69-71	-0.08 [-0.22 – 0.07] (0.308, 0.585)	-0.12 [-0.22 – -0.02] (0.015, 0.037)*	-0.00 [-0.00 – 0.00] (0.520, 0.681)	0.00 [-0.00 – 0.00] (0.663, 0.770)
PP				
36 to 43	0.05 [-0.08 – 0.17] (0.476, 0.789)	0.02 [-0.05 – 0.08] (0.589, 0.785)	-0.00 [-0.00 – 0.00] (0.967, 0.967)	-0.00 [-0.00 – 0.00] (0.908, 0.908)
43 to 53	0.00 [-0.11 – 0.11] (0.987, 0.988)	-0.02 [-0.07 – 0.04] (0.547, 0.842)	0.00 [-0.00 – 0.00] (0.392, 0.523)	0.00 [-0.00 – 0.00] (0.185 0.370)
53 to 60-64	-0.12 [-0.23 – -0.01] (0.418, 0.152)	-0.03 [-0.08 – 0.03] (0.364, 0.893)	-0.00 [-0.00 – 0.00] (0.658, 0.853)	-0.00 [-0.00 – 0.00] (0.663, 0.965)

60-64 to 69	0.12 [-0.00 – 0.24] (0.059, 0.235)	0.06 [-0.00 – 0.12] (0.051, 0.101)	-0.00 [-0.00 – 0.00] (0.537, 0.681)	-0.00 [-0.00 – 0.00] (0.431, 0.900)
69 to 69-71	0.01 [-0.10 – 0.12] (0.869, 0.871)	-0.01 [-0.07 – 0.05] (0.714, 0.7126)	-0.00 [-0.00 – 0.00] (0.683, 0.681)	0.00 [-0.00 – 0.00] (0.581, 0.770)
FHS-CVS				
36 to 53	0.16 [-0.51 – 0.19] (0.369, 0.432)	-0.11 [-0.28 – 0.07] (0.225, 0.432)	0.00 [-0.00 – 0.00] (0.313, 0.470)	0.00 [-0.00 – 0.01] (0.410, 0.470)
53 to 69	-0.03 [-0.26 – 0.19] (0.767, 0.903)	0.01 [-0.11 – 0.12] (0.906, 0.903)	0.00 [-0.00 – 0.00] (0.575, 0.903)	0.00 [-0.00 – 0.01] (0.694, 0.903)

Supplementary Table 3: Linear regression models between ACA, MCA, and PCA CBF, and SBP, DBP, and MAP. All metrics were adjusted for sex and age at scan [and for sex-interaction effects if statistically significant). An asterisk indicates associations that survived FDR correction. ACA = anterior cerebral artery; B = unstandardised beta; CBF = cerebral blood flow; DBP = diastolic blood pressure; FDR = false discovery rate; MAP = mean arterial pressure; MCA = middle cerebral artery; PCA = posterior cerebral artery; SBP = systolic blood pressure; sCoV = spatial coefficient of variation.

	B	95% CI	FDR-corrected p-value	Sex-interaction (p-value)
**ACA**				
SBP	-0.22	-0.38 – -0.06	0.02*	0.24 (0.02)*
DBP	0.33	-0.60 – -0.06	0.02*	0.19 (0.29)
MAP	-0.34	-0.58 – -0.11	0.02*	0.30 (0.06)
**MCA**				
SBP	-0.20	-0.36 – -0.05	0.02*	0.22 (0.02)*
DBP	-0.33	-0.59 – -0.07	0.02*	0.21 (0.23)
MAP	-0.33	-0.55 – -0.10	0.02*	0.288 (0.06)
**PCA**				
SBP	0.11	-0.26 – 0.02	0.12	0.13 (0.14)
DBP	-0.24	-0.47 – -0.00	0.09	0.17 (0.27)
MAP	-0.21	-0.41 – -0.01	0.09	0.19 (0.17)

Supplementary Table 4: Linear regression models between SBP, DBP, MAP, and PP at 69-71 years and log-transformed WMH volume. All metrics were adjusted for sex and age at scan [and for sex-interaction effects if statistically significant). An asterisk indicates associations that survived FDR correction. B = unstandardised beta; DBP = diastolic blood pressure; FDR = false discovery rate; MAP = mean arterial pressure; PP = pulse pressure; SBP = systolic blood pressure; WMH = white matter hyperintensities.

	**B**	**95% CI**	**p-value**
SBP	-0.00	-0.01 – 0.00	0.86
DBP	-0.00	-0.01 – 0.01	0.74
MAP	-0.00	-0.01 – 0.00	0.45
